# Recurrent Peroneal Intraneural Ganglion Cyst: Management and Review of the Literature

**DOI:** 10.7759/cureus.38449

**Published:** 2023-05-02

**Authors:** Joseph Yunga Tigre, Krisna Maddy, Emily L Errante, Meredith C Costello, Steven Steinlauf, Stephen S Burks

**Affiliations:** 1 Department of Neurosurgery, University of Miami Miller School of Medicine, Miami, USA; 2 The Miami Project to Cure Paralysis, University of Miami Miller School of Medicine, Miami, USA; 3 Department of Orthopaedic Surgery, University of Miami Miller School of Medicine, Miami, USA

**Keywords:** peroneal nerve, ganglion cyst, recurrent ganglion cyst, intraneural ganglion cyst, recurrent intraneural ganglion cyst

## Abstract

Intraneural ganglion cysts have been reported to affect the common peroneal nerve. Peroneal intraneural ganglion cysts are managed through surgical intervention. Despite surgical intervention, intraneural ganglion cysts can recur. Common intraneural ganglion cyst recurrence patterns have been proposed based on the initial surgical management of the cyst. These patterns all emphasize the importance of treatment of the proximal tibiofibular (TF) joint to reduce the risk of cyst recurrence. Although joint resection is the favored intervention in the literature, joint arthrodesis is an option for certain patients. Here, we present a case of a peroneal intraneural ganglion cyst and its recurrence in a 36-year-old male who had previously undergone surgical removal of the cyst three months prior, as well as a review of the current literature that aims to add to our current understanding of intraneural cysts.

## Introduction

Ganglion cysts typically present with a glassy, clear appearance with a jelly-like fluid and typically arise in areas under continuous mechanical stress, such as the tendon, muscle, and menisci [1]. Most cases in the literature to date are of intra-articular cystic lesions. They can present as well-defined lobulated and multiloculated lesions along the surface of the ligament, most commonly in the anterior cruciate ligament (ACL) but can also occur in the posterior cruciate ligament (PCL) [2]. Less commonly reported are intraneural ganglion cysts. These cysts occur via the outflow of synovial fluid from a joint into the epineural sheath of the articular branch of a peripheral nerve [3]. Most frequently, intraneural ganglion cysts have been reported to affect the common peroneal nerve (CPN). In a meta-analysis conducted by Desy et al. of over 600 cases of intraneural ganglion cysts, they reported 60.6% of ganglion cysts were located in the CPN [4].

Peroneal intraneural ganglion cysts present with motor deficits and peripheral neuropathy, manifesting as a foot drop with a high stepping gait and associated sensory deficits [5]. The first-line treatment for peroneal intraneural ganglion cysts is surgical intervention, usually surgical exploration, decompression of the peroneal nerve, drainage of the cyst, and obliteration of the small sensory nerve to the tibiofibular (TF) joint [6]. However, intraneural ganglion cysts’ recurrence rates are high, estimated at 11% [7]. Desy et al. identified three main types of common intraneural cyst recurrence patterns based on the initial surgical management of the intraneural ganglion cyst: Type I: parent nerve recurrence pattern occurs when surgical treatment focuses on isolated cyst decompression or resection within the parent nerve without disconnecting the articular branch and/or addressing the joint, Type II: articular branch recurrence pattern occurs following incomplete or incorrect articular branch disconnection without addressing the joint, and Type III: joint recurrence pattern occurs if the joint pathology is not initially correctly addressed, independent of correct intraneural ganglion cyst and articular branch management [7].

All three types of common intraneural ganglion cyst recurrence patterns above emphasize the importance of treating the underlying pathology in patients with recurrent intraneural ganglion cysts, as the proper intervention will reduce the risk of future cyst recurrences. Joint resection and arthrodesis are used to treat peroneal intraneural ganglia; however, resection is favored due to its simplicity over arthrodesis with no post-operative immobilization period [8]. Considering the patient’s clinical history and treatment goals, joint arthrodesis may be a preferred option for recurrent peroneal intraneural ganglion cysts. Here, we present a case of an intraneural ganglion cyst and its recurrence in a 36-year-old male who had previously undergone cyst fenestration and sectioning of the articular branch of the CPN three months prior.

## Case presentation

Initial presentation

**Figure 1:**
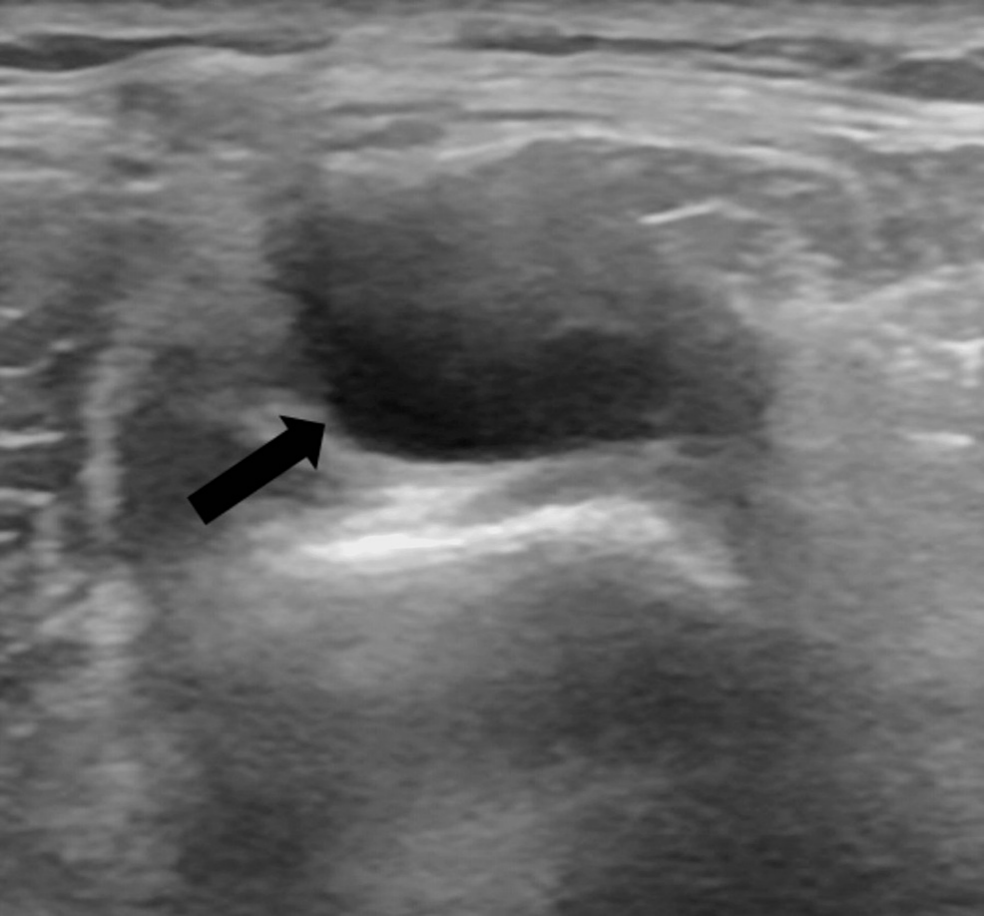
Initial ultrasound of the right lower extremity demonstrated the solid and cystic soft tissue mass (black arrow) originally found near the level of the fibular tunnel

A 36-year-old, right-handed male with no significant past medical history initially presented for evaluation of low back and right shin pain radiating to his foot. He reported that the pain began four months ago, was located at his right shin, and was intermittent. He described the pain as an ache, with a burning sensation radiating to his right foot, and associated low back pain. Gabapentin and other over-the-counter analgesics provided minimal relief. On initial physical exam, he was noted to have a right foot drop with weak right ankle dorsiflexors (2/5), long toe extensors (4/5), and decreased sensation to light touch on his right lateral calf. An ultrasound was performed that showed severe enlargement of the peroneal nerve with a cystic mass at the level of the fibular tunnel (Figure 1). An electromyography (EMG) and nerve conduction study were performed showing dysfunction of the peroneal nerve. At this time, we recommended surgical intervention. He was found to have an intraneural ganglion cyst along the right CPN into the superficial and deep branches of the nerve. He underwent neurolysis of these nerves with cyst fenestration and removal, as well as sectioning of the nerve into the proximal TF joint.

**Figure 2:**
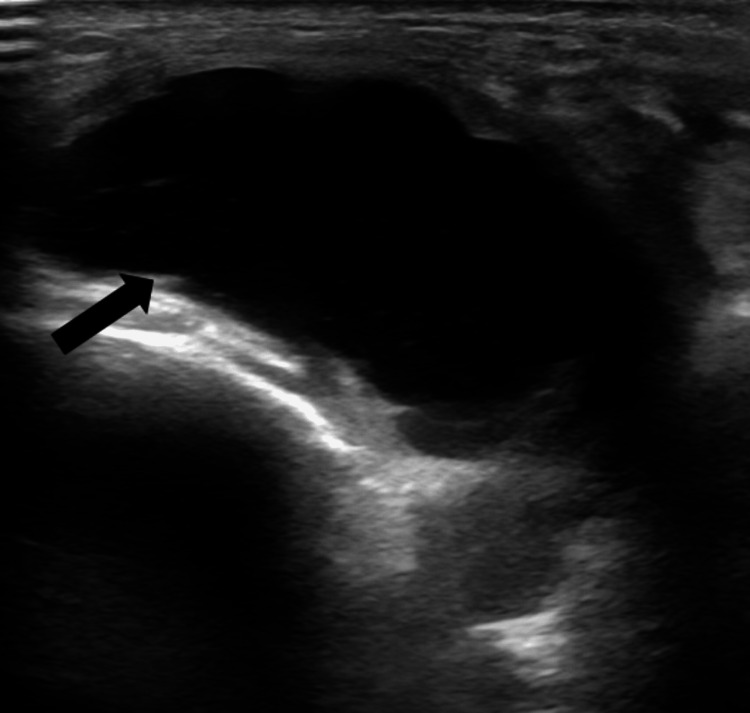
Repeat postoperative ultrasound of the right lower extremity demonstrated the recurrence of the intraneural cyst (black arrow) of the common peroneal nerve at the level of the fibular neck; additionally, the intraneural cyst can be seen in continuity with the deep peroneal nerve

**Figure 3:**
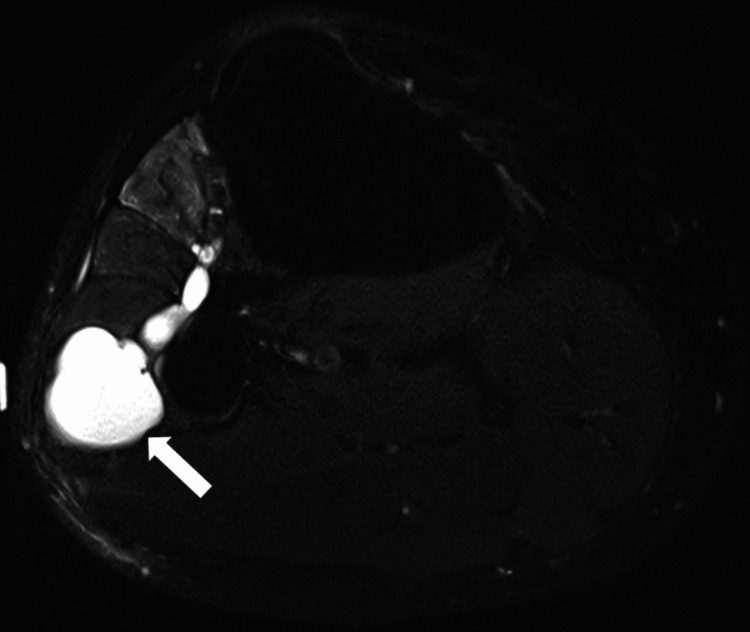
Postoperative MRI without contrast of the right lower extremity demonstrated the recurrent tubular intraneural ganglion cyst of the deep peroneal nerve and articular branch adjacent to the anterior aspect of the superior tibiofibular joint (white arrow)

Postoperatively, he did well with immediate improvement in sensation and a slight improvement in strength. Unfortunately, after about one month, he began to experience recurrent symptoms. He reported intermittent pain in the lateral aspect of his right lower leg and associated numbness radiating from his right shin to his right foot. He continued to endorse weakness in his right foot and noticed a growing “lump” at his right fibular neck. These symptoms progressed over three months, and he ultimately developed a progressive foot drop. On physical exam, tibialis anterior (TA) was 2/5, extensor hallucis longus (EHL) was 1/5, and there was decreased sensation in the right shin and inner aspect of the right foot. A repeat postoperative ultrasound of the right leg showed the recurrence of the intraneural cyst at the CPN at the level of the fibular neck (Figure 2). An MRI then showed a recurrent, tubular intraneural ganglion cyst of the deep peroneal nerve and articular branch adjacent to the anterior aspect of the superior TF joint (Figure 3). This was diagnosed as a rapid recurrence of the intraneural ganglion cyst. A combined neurological and orthopedic surgical intervention was recommended to decrease the risk of ganglion cyst recurrence.

Intervention

**Figure 4:**
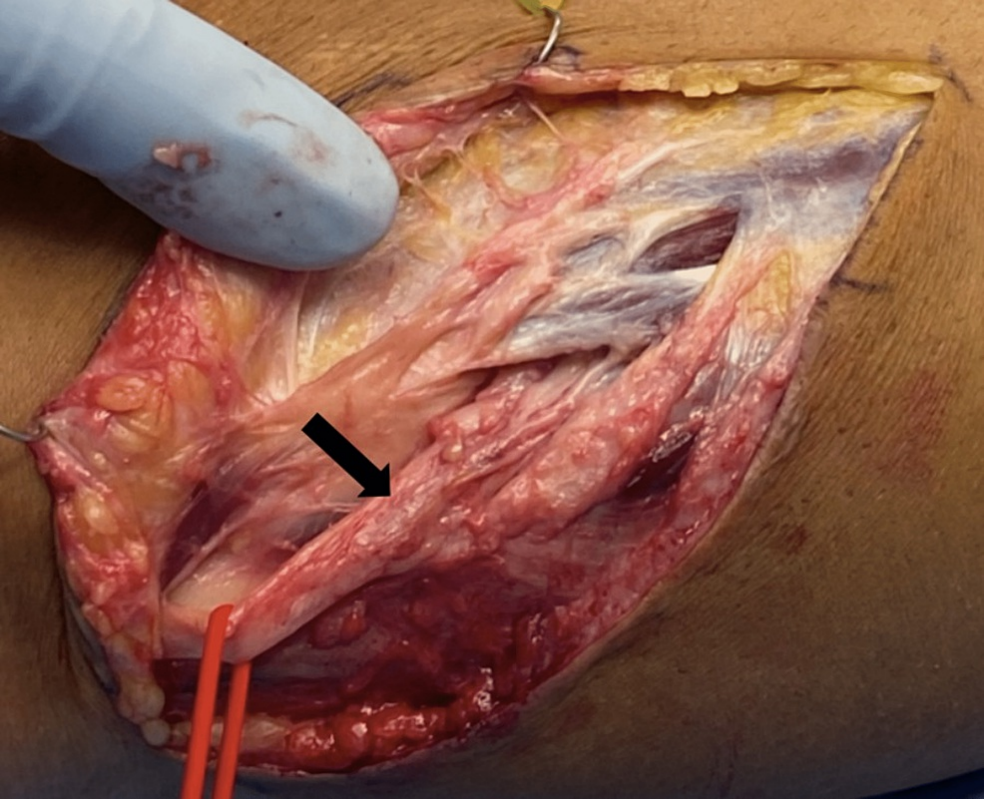
Surgical exposure of the right common peroneal nerve performed during the revision surgery The intraoperative image shows exposure of the right common peroneal nerve performed during the revision surgery. The intraneural ganglion cyst immediately drained upon exposure of the nerve. The black arrow shows the common peroneal nerve.

After discussion with the patient and his family, a mutual decision was made to proceed with right CPN revision neurolysis with decompression and removal of the ganglion cyst, as well decortication and fusion of the proximal TF joint with instrumentation. The patient was positioned semi-lateral, and the leg was prepped from mid-thigh down. A tourniquet was used intraoperatively. The CPN was identified and traced from proximal to distal to identify the branches at the fibular head. This included the identification of the superficial and deep branches and where they entered the musculature. A nerve stimulator was used, and good twitches were obtained from the superficial nerve but not the deep peroneal nerve. The recurrent ganglion cyst was found to be in the perineural space and self-decompressed upon exposure of the CPN (Figure 4). Circumferential neurolysis of the right common, superficial, and deep peroneal nerve was then performed. The TF joint was then inspected, and no remnant of the small articular branch could be identified. The orthopedic team performed fixation and fusion of the proximal TF joint with osteotomy of the fibula, as described below.

**Figure 5:**
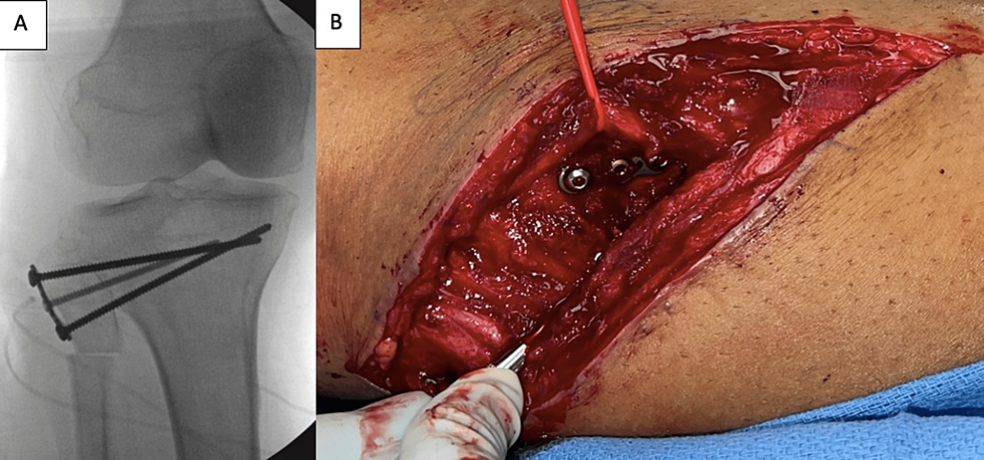
Tubular plate placement for joint arthrodesis for prevention of future recurrent intraneural ganglion cysts (A) is the intraoperative X-ray that demonstrates the placement of tubular plate and screws to compress the joint. (B) is the intraoperative image that demonstrates the placement of the tubular plate and screws on the proximal tibia to compress the joint, as well as the proximal osteotomy of the fibula.

The capsule of the proximal tibial fibular joint was identified and incised anteriorly. The joint was minimally opened and prepared. A drill hole was then made into the proximal and lateral tibial metaphysis, and bone graft was packed into this joint. The joint was then manually reduced, and a Kirschner wire (K-wire) was placed from the proximal fibula into the proximal tibia. After confirmation of correct reduction with fluoroscopic imaging, a small tubular plate with screws was placed on the proximal fibula to compress the joint (Figures 5A, 5B). An osteotomy was then performed after identifying the fibular diaphysis. Further compression of the proximal fibula to the proximal tibia was obtained to reduce rotation stress off the fusion site. Final imaging showed adequate alignment and hardware positioning. Attention was then returned to the peroneal nerve and a small amniotic membrane wrap was placed circumferentially around the common, superficial, and deep peroneal nerve. The skin was then closed, and the patient was transferred to the postoperative area.

At the six-week follow-up, there was resolution of his preoperative symptoms with stable strength. Of note, he did develop a venous thrombus and was treated with anticoagulants. 

## Discussion

The most common type of intraneural ganglion cyst is the peroneal intraneural ganglion cyst, which is said to derive from the anterior portion of the superior TF joint [9]. In more anatomical detail, peroneal interneural ganglion cysts dissect proximally along the recurrent U-shaped articular branch and typically extend into the deep peroneal component of the CPN [10]. Patients commonly present with a foot drop due to compression of the deep peroneal fascicles [10].

To our knowledge, the first cases of ganglion cysts in the peroneal nerve were reported in 1932 by Wadstein, who presented two cases of patients presenting with leg pain and confirmation of cyst appearance on imaging [11]. Following extirpation of the cysts and parts of the nerve sheath with pathological alterations, patients reported resolution of pain. At the time, he proposed that these cysts occurred secondary to trauma or degenerative changes of the nerve sheath [11].

Spinner et al. reported on the presence of a connection between the ganglion and synovial joint in several cases of surgical dissection of the ganglia in multiple anatomical locations [12]. This is summarized as Spinner’s "unified articular theory," which arose from a study of peroneal intraneural ganglion cysts. In this study, the authors examined 24 cases of peroneal intraneural ganglia. They subsequently explored the pathogenesis of peroneal cyst formation and identified a pattern of fluid dissection proximally up the articular branch within the epineurium to involve the distal peroneal nerve [12]. As for management, in addition to the decompression of the cyst, they also emphasize the importance of drainage, exploration, and ligation of the articular branch, as well as resection of the pathological superior TF joint [13]. Panwar et al. retrospectively reviewed patients with intraneural ganglion cysts and found that four patients in their review had CPN involvement [14]. They also emphasized the importance of identifying and excising the articular branch connection to the joint to prevent cyst recurrence [14]. Furthermore, Desy et al. also recommend that surgical management of intraneural ganglion cysts should be directed at the articular branch connection near the joint and/or the joint itself [7]. By correctly addressing these involved anatomical structures, the recurrence of cysts can be limited. If the cyst does recur, the mean intraneural cyst recurrence interval has been reported to be around 22 months (range, 0.5-420 months) [7].

In the common intraneural ganglion cyst recurrence patterns identified by Desy et al., underlying joint pathology, either incorrectly or not addressed, is seen in all the proposed recurrence patterns [7]. The joint connections in intraneural ganglion cysts may not be immediately identified, so initial joint resection or arthrodesis may not seem evident. This is further exemplified, as articular branch disconnection, without mention of any joint surgery, has been the most reported approach to decrease intraneural ganglion cyst recurrence. Yet, recurrence rates of intraneural ganglion cysts remain high.

Desy et al. proposed joint resection of the superior TF joint as their preferred method of treatment in cases of intraneural ganglion cyst recurrences [7]. TF joint resection should completely remove the synovium at the joint, directly addressing the source of the problem. However, Desy et al. also acknowledge that complete synovial joint resection may not be technically possible but prefer this method over joint fusion [7]. Proximal TF joint arthrodesis is an alternative option in recurrent intraneural ganglion cysts and eliminates the risk of incomplete joint resection.

In the initial surgical management of our case, despite proper ganglion cyst and articular branch management, including sectioning of the nerve to the proximal TF joint, remaining joint pathology likely led to the rapid recurrence. During revision surgery, in addition to readdressing the cyst and nerve management, proximal TF joint arthrodesis was recommended to decrease the risk of ganglion cyst recurrence. The joint synovium, likely responsible for the intraneural ganglion cyst recurrence, was thus completely resected. Through this intervention, the risk of recurrence was further reduced.

The literature on peroneal ganglion cysts and recurrence is limited, making this case an important contribution to our understanding of peripheral nerve cysts. Peroneal intraneural cysts can recur quickly and have associated joint pathology that must be addressed correctly to reduce the risk of recurrence. Joint arthrodesis may not be considered initially but could be considered for patients who present with recurrent ganglion cysts.

## Conclusions

Peroneal intraneural ganglion cysts are the most common type of intraneural ganglion cysts. Recurrence of these cysts can occur, potentially rapidly, and lead to progressive neurologic deficits. In these cases, aggressive treatment of the proximal TF joint should be performed, resection versus arthrodesis. Joint fusion potentially presents a more robust option as it limits the risk of incomplete synovial membrane removal.

## References

[1] Zantop T, Rusch A, Hassenpflug J, Petersen W (2003). Intra-articular ganglion cysts of the cruciate ligaments: case report and review of the literature. Arch Orthop Trauma Surg.

[2] Durante JA (2009). Ganglion cyst on the posterior cruciate ligament: a case report. J Can Chiropr Assoc.

[3] Vidoni A, McLoughlin E, James SL, Botchu R (2020). Intra-neural ganglion cyst of the lateral dorsal cutaneous nerve: an uncommon cause of lateral ankle pain. J Ultrasound.

[4] Desy NM, Wang H, Elshiekh MA, Tanaka S, Choi TW, Howe BM, Spinner RJ (2016). Intraneural ganglion cysts: a systematic review and reinterpretation of the world's literature. J Neurosurg.

[5] Bucher F, Maerz V, Obed D, Vogt PM, Weyand B (2022). Intraneural ganglion of the peroneal nerve-a rare cause of pediatric peroneal nerve palsy: a case report. European J Pediatr Surg Rep.

[6] Lisovski V, Minderis M (2019). Intraneural ganglion cyst: a case report and a review of the literature. Acta Med Litu.

[7] Desy NM, Lipinski LJ, Tanaka S, Amrami KK, Rock MG, Spinner RJ (2015). Recurrent intraneural ganglion cysts: pathoanatomic patterns and treatment implications. Clin Anat.

[8] Spinner RJ, Desy NM, Rock MG, Amrami KK (2007). Peroneal intraneural ganglia. Neurosurg Focus.

[9] de Sèze MP, Rezzouk J, de Sèze M (2005). Anterior innervation of the proximal tibiofibular joint. Surg Radiol Anat.

[10] Spinner RJ, Mokhtarzadeh A, Schiefer TK, Krishnan KG, Kliot M, Amrami KK (2007). The clinico-anatomic explanation for tibial intraneural ganglion cysts arising from the superior tibiofibular joint. Skeletal Radiol.

[11] Wadstein T (1932). Two cases of ganglia in the sheath of the peroneal nerve. Acta Orthop Scand.

[12] Spinner RJ, Atkinson JL, Tiel RL (2003). Peroneal intraneural ganglia: the importance of the articular branch. A unifying theory. J Neurosurg.

[13] Spinner RJ, Atkinson JL, Scheithauer BW (2003). Peroneal intraneural ganglia: the importance of the articular branch. Clinical series. J Neurosurg.

[14] Panwar J, Mathew A, Thomas BP (2017). Cystic lesions of peripheral nerves: are we missing the diagnosis of the intraneural ganglion cyst?. World J Radiol.

